# Decreased intracellular water is associated with sarcopenic obesity in chronic haemodialysis patients

**DOI:** 10.1186/s12877-023-04357-4

**Published:** 2023-10-06

**Authors:** Maolu Tian, Jing Yuan, Fangfang Yu, Pinghong He, Qian Zhang, Yan Zha

**Affiliations:** 1 Department of Nephrology, Guizhou Provincial People’s Hospital, Guiyang, China; 2 NHC Key Laboratory of Pulmonary Immunological Disease, Guizhou Provincial People’s Hospital, #83, Zhongshan Road, Nanming District, Guiyang, Guizhou 550002 China; 3 Medical College, Guizhou University, Guiyang, China

**Keywords:** Intracellular water, Haemodialysis, Sarcopenic obesity

## Abstract

**Objective:**

To explore the association between intracellular water (ICW) and sarcopenic obesity in patients undergoing chronic haemodialysis (HD).

**Methods:**

A multicentre, cross-sectional study of 3354 adult chronic HD patients was conducted in 20 haemodialysis centres from June 1, 2021, to August 30, 2021. The diagnosis of sarcopenic obesity was made according to the revised Asian Working Group’s definition of sarcopenia combined with obesity per the body fat percentage definition. Body composition was evaluated by a body composition monitor using bioimpedance spectroscopy. Multiple logistic regression models, stratified analyses, interactive analyses, and receiver-operating characteristic analyses were conducted.

**Results:**

A total of 752 patients were diagnosed with sarcopenic obesity among 3354 participants. The patients were grouped by sex-specific ICW median levels, and the prevalence of sarcopenic obesity was significantly higher in the low ICW group than in the high ICW group (41.3%vs 3.0%). Decreased ICW was significantly associated with sarcopenic obesity. The association remained statistically significant even after adjusting for dialysis vintage, age, body mass index, biochemical indicators, and various medical histories. The odds ratios of the low ICW group were much higher than those of the high ICW group in both males and females (*P* for trend < 0.001). The association was stable across subgroups, and the interaction analysis showed that age, body mass index and history of diabetes had interactive roles in the association between ICW and sarcopenic obesity (*P* for interaction < 0.05). Furthermore, the ICW cut-off values for identifying sarcopenic obesity were 19.1 kg and 14.5 kg for males and females, respectively.

**Conclusion:**

Decreased ICW was an independent risk factor for sarcopenic obesity in chronic HD patients. The measurement of ICW by bioimpedance spectroscopy might be a non-invasive and valid means for identifying the risk of future sarcopenic obesity in HD patients.

## Introduction

Sarcopenic obesity is characterized by age-related changes in body composition, including increased fat mass and decreased muscle mass, muscle strength and physical performance [1]. This condition has recently attracted increased interest in both research and clinical practice because of its negative impact on patient-centred outcomes [2–7]. Compared to patients with either sarcopenia or obesity alone, patients with sarcopenic obesity have increased risks of negative health-related outcomes such as frailty, comorbidities and mortality, leading to significantly higher health care costs [3, 4, 7–13]. One recent study suggested that the combination of sarcopenia and obesity has a synergistic effect on the occurrence of incident chronic kidney disease (CKD) in patients with type 2 diabetes [14]. Several molecular mechanisms, including inflammation and oxidative stress, insulin resistance, proteostasis imbalance, cellular senescence, and mitochondrial dysfunctions have been linked to the occurrence of sarcopenic obesity [15, 16]. Muscle fat infiltration, also known as myosteatosis, refers to any lipid deposition in the skeletal muscle and is a marker of muscle quality; a muscle with higher fat deposition has lower contraction capacity to produce force per unit of muscle mass [17, 18]. Myosteatosis has been associated with inflammation, physical inactivity, metabolic abnormalities, cardiovascular disease and increased mortality [19, 20].

Individuals with CKD exhibit the typical characteristics of elderly individuals, regardless of their chronological age, mostly due to toxin accumulation, chronic inflammation, and hormone imbalance [21]. Sarcopenic obesity occurs substantially more often in people with CKD than in those without CKD [22], and its prevalence is reported to be 3.8%-50% according to different diagnostic criteria, demographic characteristics, and cut-off values [23–29]. In haemodialysis (HD) populations, sarcopenic obesity is also independently linked to unfavorable prognoses such as poor gait performance, frailty, poor quality of life, increased cardiovascular diseases and peripheral arterial diseases, and high mortality [23, 30–34]. Sarcopenic obesity has also been found to strongly contribute to a worse clinical prognosis compared with either sarcopenia or obesity alone in CKD patients [35–37].

Sarcopenic obesity is common in patients with CKD and seriously endangers patient health. However, its early stages do not receive sufficient attention, which leads to a delay in diagnosis and treatment. Therefore, patients should undergo screening to facilitate early detection and care to prevent its progression. Intracellular water (ICW), a marker of cell hydration, was reported to reflect muscle mass and function in both elderly individuals and athletes [38, 39]. It is also well known that the water content of fat tissue is significantly lower than that of lean tissue, and the decrease in ICW is likely to be accompanied by an increase in fat tissue. We proposed the hypothesis that reduced ICW is associated with the occurrence of sarcopenic obesity. Therefore, this multicentre study aimed to assess whether ICW can be used as a valuable marker for identifying sarcopenic obesity in an HD population.

## Methods

### Study design and participants

**Fig. 1:**
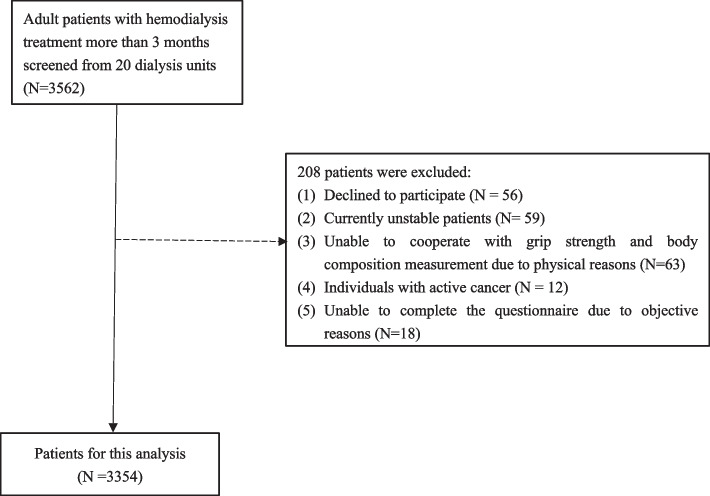
The flow chart of participant screening

A multicentre, cross-sectional study in 20 dialysis units of tertiary general hospitals in Guizhou Province was conducted from June 1, 2021, to August 30, 2021. All adult haemodialysis patients, who received 4-h bicarbonate-based dialysis treatment thrice weekly for at least 3 months or longer, were invited to participate in our study. Exclusion criteria were as follows: (i) currently unstable patients, defined as patients who were acutely ill or hospitalized at the time of the assessment; (ii) patients with any physical deformities or those with contraindications such as implanted or external electronic devices, metallic implants that hindered the measurement of body composition; (iii) inability to complete questionnaire due to language incompatibility, visual or auditory disability; (iv) patients who did not cooperate with performing muscle performance tests; (v) participants who did not have routine blood tests in the past three months preceding the study; (vi) individuals with active cancer. The flow chart of patient screening is shown in Fig. 1. Ultimately, 3354 patients were included in our analysis.

### Definition of sarcopenic obesity

Sarcopenic obesity is defined as the coexistence of sarcopenia and obesity. The diagnosis of sarcopenia was based on the revised Asian Working Group’s definition of sarcopenia (AWGS 2019) [40]. Patients with low muscle mass (defined as appendicular skeletal muscle index (ASMI) < 7 kg/m^2^ in men and < 5.7 kg/m^2^ in women) together with low muscle strength (defined as HGS < 26 kg in men and < 18 kg in women) were diagnosed with sarcopenia. The ASMI was calculated as appendicular skeletal muscle mass (ASM) in weight (kg)/height (m^2^), and the ASM was calculated according to an equation developed in Asian chronic HD patients recently [40]. Obesity was defined as a body fat percentage (BFP) ≥ 25% in men and ≥ 35% in women according to WHO recommendation, and these BFP cutoff values are also commonly used in CKD patients [41–45].

### Body composition measurement

Measurements of body composition were performed using a portable whole-body bioimpedance spectroscopy device, Body Composition Monitor (BCM, Fresenius Medical Care, Bad Homburg, Germany). The BCM measures body composition by analyzing the electrical responses at 50 different frequencies from 5 to 1000 kHz. The accuracy of BCM has been validated against the gold-standard methods [46, 47], and accumulating evidence suggests that body composition, as determined by the BCM, is a key predictor of survival in chronic HD patients [48, 49]. The measurement was carried out approximately 30 min before the HD session by a well-trained renal physician and a dialysis nurse, with four conventional electrodes being placed on the patient lying in supine position: two on the hand and two on the foot contralateral to the vascular access. In order to reduce operator variability, according to the manufacturer’s instructions, all tests were performed by the same operators. intracellular water, extracellular water, total body water and body fat mass were retrieved from the BCM software. BFP was calculated as fat mass divided by body weight.

### Anthropometric measurements

Body weight was measured with an electronic scale while participants were wearing lightweight clothing. Standing height was assessed using a stadiometer with participants barefoot and standing erect. BMI was calculated as body weight in kilogram divided by height in meter squared. Hand grip strength (HGS) was measured in the dominant or non-fistula hand, using CAMRY® dynamometer with a precision of 0.1 kg. The patients sitting with their arms bent at an angle of 90º on a horizontal level held the tool with the fingers around it. Three measures were taken with 30 s of rest between each test and the maximum score was adopted for the study.

### Collection of other variables

All the patients' individual information including sociodemographic data, comorbid conditions, drug usage, dialysis duration, dialysis mode, blood pressure, and biochemistry indexes was collected from the electronic medical record system, dialysis run sheets and a face-to-face interview with a predesigned questionnaire. Patients with a high school level were defined as individuals who had received more than nine years of schooling education. We collected the above data from each center within a strict quality-control framework, and further checked them to ensure the accuracy of our database. In this study, Stroke included hemorrhagic and ischemic stroke with a clinical history, confirmed by brain imaging test such as computed tomography or magnetic resonance. Cardiovascular disease (CVD) was defined as coronary artery disease (angina, myocardial infarction, percutaneous coronary intervention or coronary bypass grafting), congestive heart failure requiring hospitalization, arrhythmia (atrial fibrillation or other arrhythmia) and peripheral vascular disease (limb claudication needing percutaneous angioplasty and/or bypass grafting as treatment).

### Statistical analysis

Continuous data with a skewed distribution were expressed as the medians and interquartile ranges. Categorical data were expressed as numbers and percentages. There has not been a uniform standard of ICW to define abnormal cellular hydration status as it might change with sex, race, detection methods and so on in HD patients. Therefore, this study used the sex-specific ICW median as surrogate marker for distinguishing between "High” or “Low” ICW level. We split patients into two groups according to sex-specific ICW median levels, resulting in High and Low groups with almost equal group size. To compare the patient characteristics of the two groups, Mann–Whitney U tests or Chi-square tests was used. Multivariable logistic regression analysis was performed to assess odds ratios (ORs) and 95% confidence intervals (CIs) for the association of ICW with risk of sarcopenic obesity, with ICW as continuous variables, or the high ICW groups as the reference. Variables that were statistically significant by univariate analysis were adjusted in the multivariate logistic regression models. To evaluate the robustness of the primary results, the subgroup analysis was performed to explore the potential effect modification by age (< 65 years; and ≥ 65 years), BMI (< 25 kg/m^2^; and ≥ 25 kg/m^2^), history of CVD (yes or no), and history of diabetes (yes or no). The potential interactions were evaluated across subgroups. The receiver operating characteristic (ROC) curve analysis was conducted to evaluate the performance and cutoff value of ICW. MedCalc software (version 19.0.4) was used to perform ROC curve analysis. The statistical packages R (http://www.r-project.org; version4.0.1) was used to perform subgroup analysis. Other statistical analyses were performed using SPSS software (version 26.0; SPSS Inc., Chicago, IL, USA). A two-sided *p* < 0.05 was considered statistically significant.

## Results

### Characteristics of patients according to ICW median levels

A total of 3354 participants were included in our final analysis. Clinical characteristics of the enrolled patients are shown in Table 1 for all subjects and the low vs high ICW group. Of the 3354 participants recruited for this study, 2022 (60.3%) were men; the median age of the participants was 57 years (interquartile range: 46.8–68). A total of 752 (22.4%) participants were diagnosed as having sarcopenic obesity according to the criteria used in this study. The median ICW for men was 19.6 kg, and 15.3 kg for women. The prevalence of sarcopenic obesity in patients with low ICW was significantly higher than that in high ICW group (41.3% vs 3.0%). There were statistically reduced trends toward education level, albumin, SBP, DBP, BMI, ASMI and HGS with decreased ICW. Conversely, age, CRP, BFP, and dialysis vintage increased with decreasing ICW levels. Participants were more likely to have a history of diabetes, stroke and CVD with decreasing ICW levels. While usage of L-carnitine, serum lipids level, hemoglobin, platelet, and white blood cell were similar across two groups (*p* > 0.05). In order to remove medium and large molecular toxins, ensuring the dialysis adequacy of patients, most patients in this study also received two other dialysis modes regularly on the basis of haemodialysis (HD), namely hemodiafiltration (HDF) and hemoperfusion (HP). However, there was also no significant difference in the percentage of patients receiving HDF and HP between the two groups.

**Table 1:** Patients’ characteristics grouped by intra-cellular water median levels

**Characteristics**	**Total**(*n* = 3354)	**High group** Men > 19.6 kg (*n* = 995) Women > 15.3 kg (*n* = 656)	**Low group** Men ≤ 19.6 kg (*n* = 1027) Women ≤ 15.3 kg (*n* = 676)	*p* value
Age (years)	57.0 (46.8, 68.0)	54.0 (44.0, 64.0)	60.0 (50.0, 72.0)	< .001
Education status (high school) (%)	1051 (31.3)	545(33.0)	506 (29.7)	0.04
Diabetes (%)	983 (29.3)	421(25.5)	562 (33.3)	< .001
CVD (%)	1551 (46.2)	714(43.2)	837 (49.1)	0.001
History of stroke (%)	410 (12.2)	163 (9.9)	247 (14.5)	< .001
HDF treatment (%)	2852 (85.0)	1404(85.3)	1443 (84.7)	0.621
HP treatment (%)	2683 (80.0)	1337(81.0)	1346 (79.0)	0.159
Usage of L-carnitine (%)	2786 (83.1)	1366 (82.7)	1420(83.4)	0.619
Dialysis vintage (months)	64.0 (44.0, 96.0)	61.0 (41.0, 92.0)	69.0 (46.0, 105.0)	< .001
BMI (kg/m^2^)	22.8 (20.6, 25.2)	23.6(21.4, 26.3)	22.0 (20.0, 24.3)	< .001
ASMI (kg/m^2^)	6.4 (5.6, 7.1)	7.0 (6.3, 7.6)	5.9 (5.2, 6.4)	< .001
HGS (kg)	20.0 (14.0, 26.6)	22.0 (16.0, 30.0)	18.0 (13.0, 23.0)	< .001
BFP (%)	25.0 (17.3, 31.6)	20.8 (14.5, 27.9)	28.0 (21.4, 35.0)	< .001
SBP (mmHg)	136.0 (123.0, 149.0)	137.0(125.0,150.0)	135.0 (121.0,148.0)	< .001
DBP (mmHg)	77.0 (68.0, 86.0)	78.0 (70.0, 88.0)	75.0 (67.0, 85.0)	< .001
Hemoglobin (g/L)	110.0 (97.0, 123.0)	109.0 (97.0, 122)	111.0 (98, 124.0)	0.218
WBC (× 10^9/L)	6.1 (5.0, 7.4)	6.1 (5.0, 7.3)	6.0 (4.9, 7.4)	0.661
PLT (× 10^9/L)	171.0 (133.0,214.0)	173.0 (136.0, 216.0)	166.0 (133.0, 213.0)	0.051
Albumin (g/L)	40.0 (37.6, 42.6)	40.5(38.1, 43.0)	39.6 (37.2, 42.1)	< .001
Total cholesterol (mmol/L)	3.8 (3.2, 4.5)	3.8 (3.2, 4.5)	3.8 (3.2, 4.5)	0.892
Triglyceride (mmol/L)	1.5 (1.1, 2.3)	1.5 (1.1, 2.4)	1.5 (1.0, 2.2)	0.629
LDL-c(mmol/L)	2.1 (1.6, 2.6)	2.1 (1.6, 2.6)	2.1 (1.6, 2.6)	0.843
CRP (mg/L)	3.0 (1.4, 7.8)	2.8 (1.3, 6.7)	3.3 (1.5, 8.8)	< .001
Sarcopenic obesity (%)	752 (22.4)	49 (3.0)	703 (41.3)	< .001

*CVD* cardiovascular disease, *HDF* hemodiafiltration, *HP* hemoperfusion, *ASMI* appendicular skeletal muscle index, *HGS* handgrip strength, *BFP* body fat percentage, *SBP* systolic blood pressure, *DBP* diastolic blood pressure, *WBC* white blood cell, *PLT* platelet, *LDL-c* low density lipoprotein cholesterol, *CRP* C-reactive protein, *BMI* body mass index

### Association between ICW and sarcopenic obesity

In univariate logistic regression analysis, these parameters including ICW, dialysis vintage, age, sex, usage of L- carnitine, history of diabetes, history of CVD, history of stroke, hemoglobin, albumin, SBP, DBP, triglyceride, BMI and CRP were statistically correlated with sarcopenic obesity (*p* < 0.05). With the high ICW group as the reference or ICW as continuous variables, multivariate-adjusted ORs and 95% CIs for sarcopenic obesity were summarized in Table 2. When regarded as continuous variables, higher ICW was a protective factor for the occurrence of sarcopenic obesity both in male and female (OR, 0.57 and 0.48 respectively). Individuals in low ICW group was 21.9-fold for men and 20.2-fold for women as likely to have sarcopenic obesity compared to those in the high ICW group after adjusting for other above potential confounders.

**Table 2:** Logistic regression analyses for sarcopenic obesity according to intra-cellular water level

	Model 1		Model 2		Model 3	
Intra-cellular water	OR (95%CI)	*P*	OR (95% CI)	*P*	OR (95% CI)	*P*
***Continuous variable***						
Male	0.58(0.55–0.61)	< .001	0.58(0.55–0.62)	< .001	0.57(0.53–0.60)	< .001
Female	0.47(0.42–0.52)	< .001	0.49(0.44–0.55)	< .001	0.48(0.42–0.54)	< .001
***Categorical variable***						
Male, Low vs High	24.0(17.0–33.9)	< .001	20.2(14.2–28.7)	< .001	21.9(15.2–31.6)	< .001
Female, Low vs High	25.0(13.5–46.4)	< .001	22.3(12.0–41.6)	< .001	20.2(10.5–38.9)	< .001

Model 1, Unadjusted model

Model 2, adjusted for age and dialysis vintage

Model 3, adjusted for above + history of diabetes, CVD, stroke + usage of L- carnitine + BMI + albumin + hemoglobin + SBP + DBP + CRP + triglyceride

*Note*. Patients with intra-cellular water above the median was considered as a reference group

*ORs* Odds ratios, *CI* confidence interval, *CVD* cardiovascular disease, *SBP* systolic blood pressure, *DBP* diastolic blood pressure, *CRP* C-reactive protein, *BMI* body mass index

### Subgroup analyses of correlations between ICW and sarcopenic obesity

**Fig. 2:**
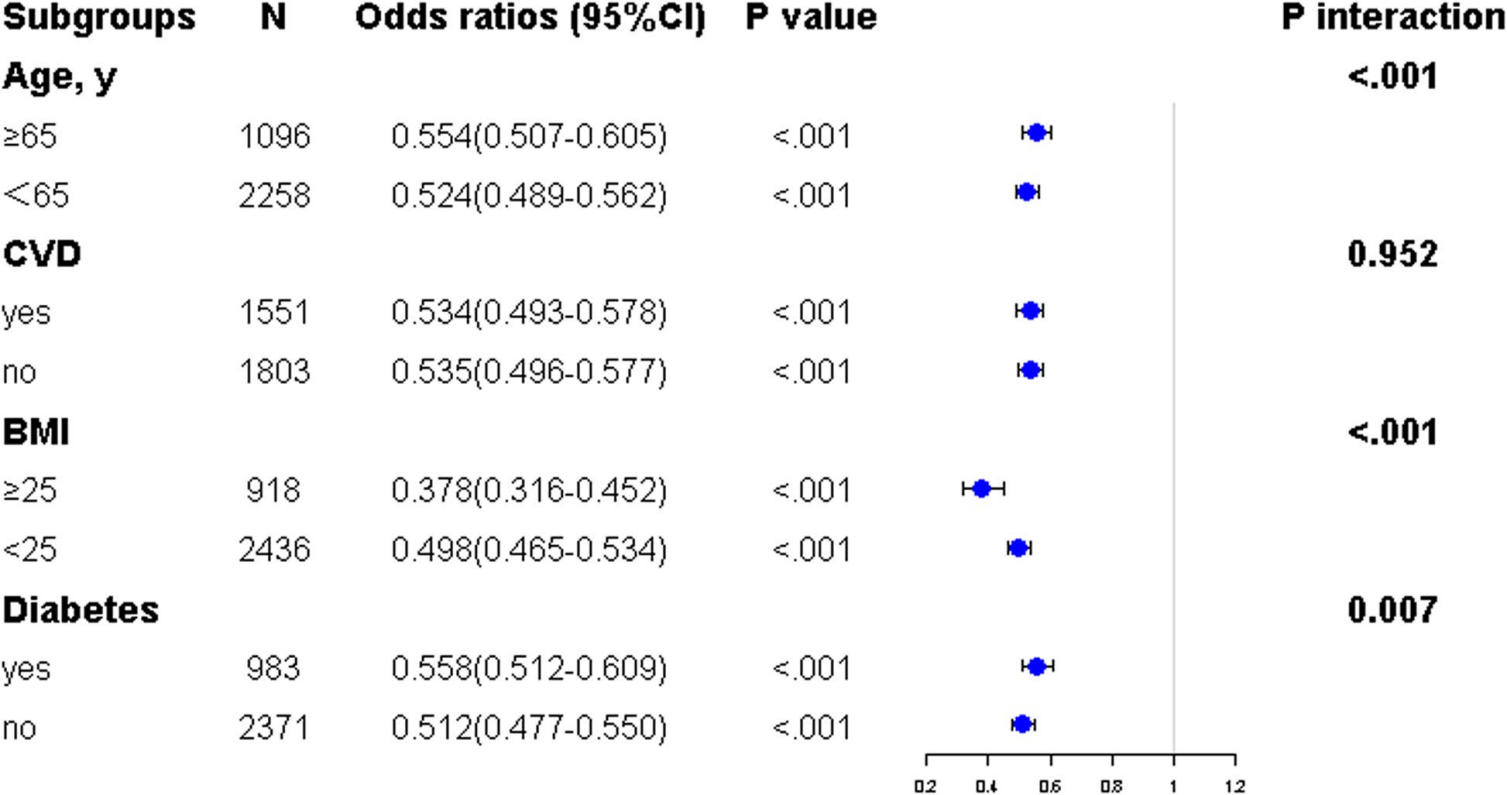
Subgroup analyses of the association between intra-cellular water and sarcopenic obesity stratified by age, diabetes, body mass index, and cardiovascular disease. Notes: Odds ratios (ORs) were calculated after adjusting for age, sex, history of diabetes, cardiovascular disease, history of stroke, usage of L-carnitine, hemoglobin, albumin, systolic blood pressure, diastolic blood pressure, body mass index, triglyceride and C-reactive protein if not stratified

To further test the robustness of association between ICW and risk of sarcopenic obesity in different subgroups, subgroup analyses of multivariable logistic regression were performed (ICW as continuous variables) in various subgroups (Fig. 2). Overall, after adjusting for age, sex, dialysis vintage, history of stroke, history of diabetes, history of CVD, usage of L-carnitine, albumin, SBP, DBP, hemoglobin, triglyceride, BMI and CRP except for the stratified variable, the association between ICW and sarcopenic obesity stably persisted across subgroups stratified by age, diabetes, BMI, and CVD. The interaction analysis showed that age, BMI and diabetes had interactive roles in the association between ICW and sarcopenic obesity (all *p* for interaction < 0.05).

### ROC analysis for ICW to identify patients with risk of sarcopenic obesity

**Fig. 3:**
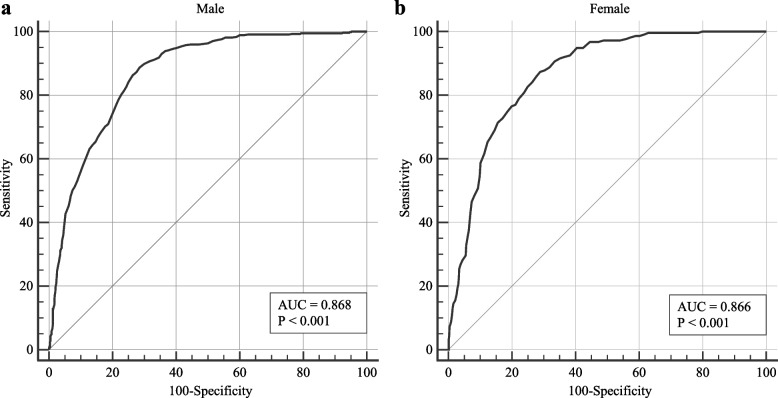
The receiver operating characteristic curve of intra-cellular water for identifying patients with high risk of sarcopenic obesity. **a** Male, **b** Female

In male patients, the area under the ROC curve (AUC) was 0.868, with a sensitivity of 89% and a specificity of 71%, and the cutoff point was 19.1 kg (Fig. 3). In female, the AUC was 0.866, with a sensitivity of 87% and a specificity of 71%, and the cutoff point was 14.5 kg (Fig. 3). Therefore, ICW with different cutoffs had similar recognition value for sarcopenic obesity in male and female.

## Discussion

To our knowledge, this is the first multicentre study to investigate the association between intracellular water and sarcopenic obesity in chronic HD patients. Our study showed that the prevalence of sarcopenic obesity increased with decreasing intracellular water. Intracellular water was associated with sarcopenic obesity in chronic HD patients independent of dialysis vintage, age, history of CVD, history of stroke, history of diabetes, usage of L- carnitine, SBP, DBP, haemoglobin, albumin, triglyceride, BMI and CRP. Although age, BMI, and history of diabetes had interactive roles in the association between intracellular water and sarcopenic obesity, the association stably existed across subgroups. The ROC analysis indicated that different cut-offs for ICW had similar recognition values for sarcopenic obesity in male and female patients. In addition, our data suggested that there was no significant association between extracellular water and sarcopenic obesity.

Although the exact mechanisms responsible for the association between low ICW and sarcopenic obesity are still unclear, it is speculated that a shared pathogenesis, such as malnutrition, chronic inflammation and oxidative stress, ageing, and insulin insensitivity might contribute to both low ICW and sarcopenic obesity. In our study, compared with patients in the high ICW group who were less likely to have sarcopenic obesity (3.0% vs 41.3%), patients in the low ICW group had higher CRP levels; lower serum albumin; lower blood pressure; longer dialysis vintage; relatively advanced ages; and more complications, including DM, CVD and stroke, indicating that lower ICW and sarcopenic obesity might share a common pathogenesis.

ICW is a metabolic signal that regulates cell function, with cell swelling stimulating anabolism and cell shrinkage stimulating catabolism and protein degradation [50, 51]. When cells are dehydrated, enzyme activity is altered, and the damaging effects on the cytoskeleton and the nucleus [52] lead to apoptosis and cell death. Elderly individuals with a lower ICW had a worse functional performance and increased frailty risk, suggesting a harmful effect of cell dehydration [39, 53, 54]. As with the age-related ICW decline, ICW significantly decreases over time in HD patients. Lower ICW levels have been reported to be associated with worse dialysis tolerance, a higher prevalence of dizziness, and increased death risk in HD patients [55]. Nevertheless, this study first investigated the relationship between ICW and sarcopenic obesity and identified appropriate ICW cut-off values for the identification of sarcopenic obesity. Of course, further studies are required to verify the validity and reliability of the calculated values in other populations. Among the treatments for sarcopenic obesity, though exercise therapy, including intradialytic and interdialytic exercises, is effective to some extent in improving sarcopenic obesity [56–60], the curative effect is limited. A study suggested that melatonin combined with exercise training could attenuate sarcopenic obesity-induced skeletal muscle dysfunction, at least in part, through preserving the satellite cell pool by inhibiting cellular senescence and attenuating mitochondrial dysfunction [61]. Another study showed that BAM15-mediated mitochondrial uncoupling could prevent sarcopenic obesity in aged mice via an inflammation-mitochondria- endoplasmic reticulum axis [62]. In view of the high prevalence and harmfulness of sarcopenic obesity, more effective drugs are expected to be used in the clinic as soon as possible.

The strengths of the current study were the relatively large sample size, multicentre representation, subgroup analyses, and detailed ascertainment of potential confounders increasing the reliability of the results. However, several limitations should also be considered. First, the study was based on a cross-sectional design, so it is not possible to determine causal relationships. Second, all patients in the present study came from a province southwestern China, thereby raising the possibility of selection bias. Third, some unmeasured and undetected confounders cannot be excluded. Fourth, sarcopenic obesity was diagnosed based on bioimpedance spectroscopy. Although imaging technologies are the most valid and reliable clinical methods for measuring body composition, they are radioactive, expensive to use in clinical practice and inconvenient to be use in epidemiological studies. Recently, bioimpedance analysis has been recognized as an ideal tool for assessing body composition in both the general population and patients with kidney disease [63, 64].

In conclusion, lower intracellular water was associated with an increased risk of sarcopenic obesity in both male and female chronic HD patients. Our findings suggest that the measurement of intracellular water by bioimpedance spectroscopy might be a simple and valid means for identifying the risk of future sarcopenic obesity in haemodialysis patients.

## Data Availability

The datasets used and/or analyzed during the current study available from the corresponding author on reasonable request.
